# Increasing venoarterial extracorporeal membrane oxygenation flow negatively affects left ventricular performance in a porcine model of cardiogenic shock

**DOI:** 10.1186/s12967-015-0634-6

**Published:** 2015-08-15

**Authors:** Petr Ostadal, Mikulas Mlcek, Andreas Kruger, Pavel Hala, Stanislav Lacko, Martin Mates, Dagmar Vondrakova, Tomas Svoboda, Matej Hrachovina, Marek Janotka, Hana Psotova, Svitlana Strunina, Otomar Kittnar, Petr Neuzil

**Affiliations:** Cardiovascular Center, Na Homolce Hospital, 15030 Prague, Czech Republic; Department of Physiology, First Faculty of Medicine, Charles University in Prague, Prague, Czech Republic; Faculty of Biomedical Engineering, Czech Technical University in Prague, Prague, Czech Republic

**Keywords:** Extracorporeal membrane oxygenation, Extracorporeal life support, Cardiac performance, Left ventricle, Cardiogenic shock

## Abstract

**Background:**

The aim of this study was to assess the relationship between extracorporeal blood flow (EBF) and left ventricular (LV) performance during venoarterial extracorporeal membrane oxygenation (VA ECMO) therapy.

**Methods:**

Five swine (body weight 45 kg) underwent VA ECMO implantation under general anesthesia and artificial ventilation. Subsequently, acute cardiogenic shock with signs of tissue hypoxia was induced. Hemodynamic and cardiac performance parameters were then measured at different levels of EBF (ranging from 1 to 5 L/min) using arterial and venous catheters, a pulmonary artery catheter and a pressure–volume loop catheter introduced into the left ventricle.

**Results:**

Myocardial hypoxia resulted in a decline in mean (±SEM) cardiac output to 2.8 ± 0.3 L/min and systolic blood pressure (SBP) to 60 ± 7 mmHg. With an increase in EBF from 1 to 5 L/min, SBP increased to 97 ± 8 mmHg (P < 0.001); however, increasing EBF from 1 to 5 L/min significantly negatively influences several cardiac performance parameters: cardiac output decreased form 2.8 ± 0.3 L/min to 1.86 ± 0.53 L/min (P < 0.001), LV end-systolic volume increased from 64 ± 11 mL to 83 ± 14 mL (P < 0.001), LV stroke volume decreased from 48 ± 9 mL to 40 ± 8 mL (P = 0.045), LV ejection fraction decreased from 43 ± 3 % to 32 ± 3 % (P < 0.001) and stroke work increased from 2096 ± 342 mmHg mL to 3031 ± 404 mmHg mL (P < 0.001). LV end-diastolic pressure and volume were not significantly affected.

**Conclusions:**

The results of the present study indicate that higher levels of VA ECMO blood flow in cardiogenic shock may negatively affect LV function. Therefore, it appears that to mitigate negative effects on LV function, optimal VA ECMO blood flow should be set as low as possible to allow adequate tissue perfusion.

## Background

Extracorporeal membrane oxygenation (ECMO) in the venoarterial (VA) configuration is an established method for the treatment of severe and rapidly progressing cardiogenic shock or refractory cardiac arrest [1–3]. In these critical conditions, VA ECMO may even fully substitute for cardiac pumping and pulmonary gas exchange to maintain sufficient tissue perfusion with oxygenated blood. Although randomized prospective trials confirming this have yet to be performed, several retrospective studies and case series demonstrating favourable outcomes have been published [4–6].

During peripheral VA ECMO therapy, an inflow venous cannula is inserted into the right atrium and drains blood into the extracorporeal blood pump. Blood gases are then exchanged in the membrane oxygenator and the oxygenated blood is typically returned to the descending aorta through a femoral arterial outflow cannula. This VA ECMO setting offers partial or full circulatory support; however, it may be associated with specific consequences for the failing heart. The inflow component of the VA ECMO circuit decreases preload and partially or completely unloads the right heart, whereas the outflow component increases left ventricular afterload [7]. In cases of extremely compromised left ventricle systolic function combined with increased afterload and, possibly, persisting (or increasing) bronchial arterial circulation or aortic and mitral regurgitation, the failing left ventricle becomes overloaded, although the right ventricle may be fully unloaded [7–9]. Progressive distension of the overloaded left ventricle with subsequent severe pulmonary edema is a critical condition that often requires urgent intervention (e.g., left ventricular assist device implantation) [3, 7–10].

Although it is clear that increasing extracorporeal blood flow (EBF) may increase left ventricular afterload, current data regarding the relationship among EBF, central hemodynamics and left ventricular performance are insufficient. Several experimental and clinical studies have focused on the impairment of left ventricular function during ECMO in neonates with pulmonary hypertension [11–16]; however, data in the literature regarding adult individuals with cardiogenic shock are very limited [17]. The objective of the present study was, therefore, to describe hemodynamic and left ventricular performance changes resulting from a gradual increase in EBF during VA ECMO in a porcine model of cardiogenic shock.

## Methods

The present study was approved by the Charles University 1st Medical School Institutional Animal Care and Use Committee and was performed at the Animal Laboratory, Department of Physiology, 1st Medical School, Charles University in Prague and Na Homolce Hospital, Prague, Czech Republic, in accordance with Act No 246/1992 Coll. on the protection of animals against cruelty. The investigation conforms to the Guide for the Care and Use of Laboratory Animals published by the US National Institutes of Health (NIH Publication No. 85-23, revised 1985).

### Animal model

Five female swine (*Sus scrofa domestica*, landrace × large white crossbreed), 4–5 months of age, with a mean body weight of 45 kg were included in the experiment. After a 24 h fast, general anesthesia was induced by administration of midazolam (0.3 mg/kg intramuscular) and ketamine hydrochloride (15–20 mg/kg intramuscular). Initial propofol and morphine boluses (2 mg/kg intravenous [18] and 0.1–0.2 mg/kg IV, respectively) were administered, and animals were orotracheally intubated. Continuous IV infusions of propofol (8–10 mg/kg/h) and morphine (0.1–0.2 mg/kg/h) were used to maintain anesthesia. The doses were adjusted according to physiological parameters, photoreaction, corneal and palpebral reflexes, lacrimation and spontaneous movement. At the conclusion of the experiment, potassium chloride (2 mEq/kg), in conjunction with general anesthesia, was used to euthanize the animals.

Bilateral femoral (arterial and venous) and jugular approaches were used for multiple sheath insertions using the standard percutaneous Seldinger technique. An initial rapid IV infusion of 1000 mL normal saline was given after anesthesia induction, followed by a continuous IV drip at a rate of 100–500 mL/h to reach and maintain a mean right atrial pressure of 5–7 mmHg (at an EBF of 1 L/min). An unfractionated heparin bolus (100 U/kg IV) was administered after sheath placement, followed by a continuous IV infusion of 50 U/kg/h to maintain an activated clotting time of 180–250 s. Values were monitored every hour using the Hemochron Junior + Microcoagulation System (ITC, USA).

Ventilation was provided by a Hamilton G5 ventilator (Hamilton Medical AG, Switzerland) set to the INTELLiVENT—Adaptive Support Ventilation mode. The ventilator was set to maintain an oxygen saturation (SpO_2_) of 95–99 %, and an end-tidal CO_2_ pressure of 4.8–5.6 kPa.

### ECMO

The ECMO circuit consisted of a Levitronix Centrimag console (Thoratec, USA), centrifugal pump, tubing set with HMO 70000 Adult Microporous Membrane Oxygenator with Softline Coating (MAQUET Cardiopulmonary AG, Germany) and a mechanical gas blender (Sechrist, USA). Biomedicus cannulae (Medtronic, USA) were introduced percutaneously using the standard Seldinger technique after repeated dilations of the femoral artery and vein. For the insertion of ECMO cannulas the larger diameter left or right femoral arteries and veins were used according to the preceding duplex ultrasound measurement. The venous inflow cannula (21 Fr) was inserted into the right atrium (the tip position was verified by fluoroscopy) and the femoral arterial outflow cannula (15 Fr) was inserted into the femoral artery. Blood gas parameters were monitored continuously in the blood leaving the oxygenator (CDI™ Blood Parameter Monitoring System 500, Terumo Cardiovascular Systems Corporation, USA). The oxygen/air flow was repeatedly adjusted to maintain pO_2_ and pCO_2_ in the ranges of 10–15 kPa and 4.0–6.5 kPa, respectively, in blood leaving the oxygenator. The EBF was set to 1 L/min until the start of the measurements. The circuit, console function and initial settings were controlled and directed by a perfusionist.

### Vital function and hemodynamic monitoring

Arterial pressure was measured using standard invasive methods with pressure transducers (Truwave, Edwards Lifesciences, LLC, USA) through a pigtail catheter inserted into the aortic arch. A Swan–Ganz catheter was introduced via a femoral vein to the pulmonary artery and pulmonary cardiac output (_P_CO) was measured at the end of each level of EBF. Electrocardiography, heart rate (HR), invasive blood pressures (aortic arch and jugular vein), pulse oximetry, capnometry and invasive central venous oxygen saturation were continuously monitored in all animals (Monitor Life Scope TR, Nihon Kohden, Japan; and Vigilance II, Edwards Lifesciences, USA). Brain oxygenation levels were measured using near-infrared spectroscopy (INVOS Cerebral/Somatic Oximeter, Somanetics, USA).

### Pressure–volume analysis

**Fig. 1:**
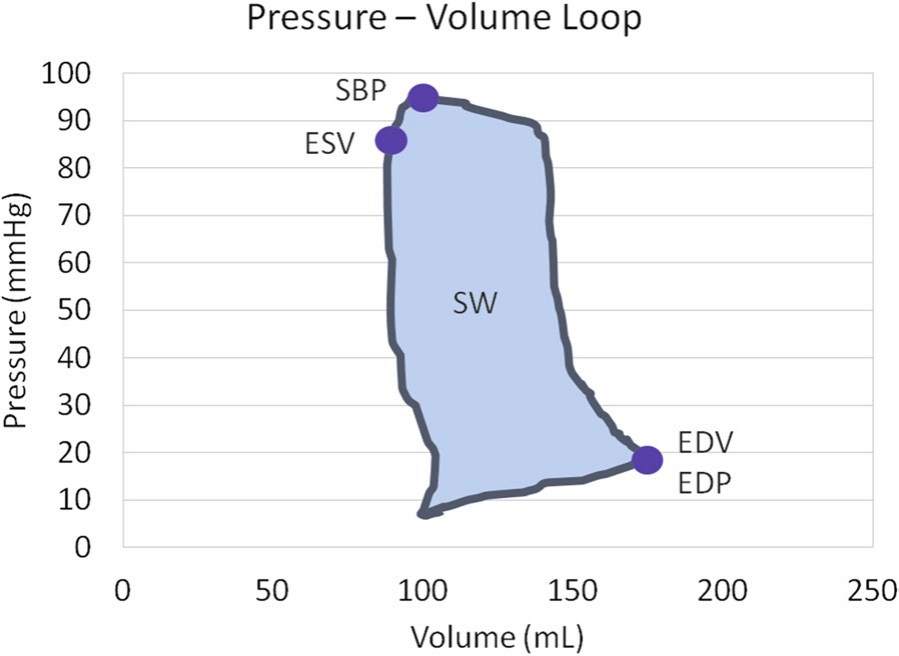
Parameters measured from pressure–volume loop. *EDP* end-diastolic pressure, *EDV* end-diastolic volume, *ESV* end-systolic volume, *SBP* systolic blood pressure, *SW* stroke work

A pressure–volume (PV) conductance catheter (Scisense 7F VSL Pigtail, Transonic, USA) was introduced into the left ventricle from the left carotid artery through the aortic valve. The catheter was connected to the PV unit (Sciense ADV 500, Transonic, USA) and operated in admittance mode. Correct positioning was assessed radiographically by confirming optimal PV loop morphology. The volume was calibrated against pulmonary thermodilution (Combo CCO catheter, Edwards Lifesciences, USA) at baseline. The PV values were recorded continually during the experiment, and data from five end-expiration loops at the end of each level of EBF were averaged and used for the analysis. Collected data included end-diastolic pressure (EDP), end-diastolic volume (EDV), systolic blood pressure (SBP), end-systolic volume (ESV) and stroke work (SW) (Fig. 1).

### Calculated parameters

Stroke volume (SV) was calculated as SV = EDV − ESV; left ventricular ejection fraction (LVEF) was calculated as LVEF = SV/EDV; left ventricular cardiac output (_LV_CO) was calculated as _LV_CO = SV × HR; recirculation minute volume (RecV) was calculated as RecV = _LV_CO − _P_CO; and recirculation fraction (RecF) was calculated as RecF = RecV/_LV_CO.

### Cardiogenic shock induction

**Fig. 2:**
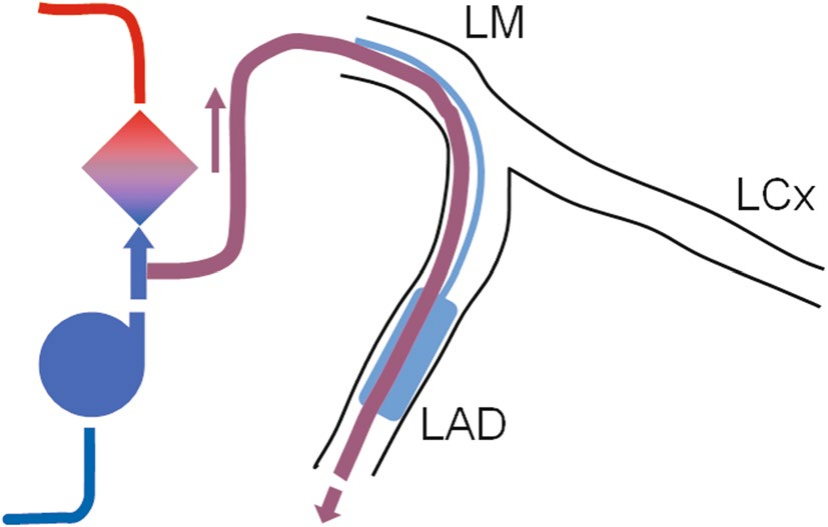
Induction of regional myocardial hypoxia through perfusion of selected coronary artery by desaturated venous blood. *LAD* left anterior descending artery, *LCx* left circumflex artery, *LM* left main artery

The most frequently used model of acute cardiogenic shock in large animals is coronary artery occlusion, ligation or embolization with subsequent myocardial infarction [19–21]. However, this approach is associated with a very high acute mortality rate [19–21]. Therefore, we developed an alternative model of myocardial hypoxia. Coronary angiography was performed and, according to the specific coronary anatomy in each animal, the largest left main coronary artery branch (left anterior descending artery or left circumflex artery) was identified. Two coronary guide wires were then introduced into the selected vessel. The first wire was used for the placement of a balloon catheter and the second for introduction of an over-the-wire export catheter (Medtronic, USA) with the tip distal to the end of balloon. The entry of the Export catheter was connected to the ECMO circuit between the pump and oxygenator (Fig. 2). After inflation of the balloon, the coronary artery was perfused with venous blood at a rate of approximately 40 mL/min. Cardiogenic shock with signs of tissue hypoperfusion was defined as a drop in systolic blood pressure to <100 mmHg and at least one of the following criteria: increase in blood lactate to >2.0 mmol/L; decrease of mixed venous oxygen saturation to <50 %; or fall in brain tissue oxygen saturation to <50 %. In cases in which the above procedure was insufficient to cause cardiogenic shock, an additional balloon catheter was introduced into the periphery of the second left main coronary artery branch and myocardial infarction was induced in the respective area by inflation of the balloon.

### Experimental protocol

After insertion of all catheters and establishment of ECMO, the animals were stabilized for 10 min. Cardiogenic shock was then induced using the above mentioned procedure. If necessary, defibrillation was performed and amiodaron 150 mg was administered IV to ensure myocardial electrical stabilization. After development of signs of tissue hypoperfusion (approximately 60–90 min) and an additional 10 min of stabilization, the EBF was gradually increased by 1 L/min every 5 min. Once an EBF level of 5 L/min was reached, it was maintained for 10 min; subsequently, the EBF was gradually decreased every 5 min by 1 L/min. At an EBF level of 1 L/min, the animals were again stabilized for 10 min and a second cycle of stepwise EBV increase and decrease was subsequently performed (Fig. 2). Measurements from the end of each 5 min interval were averaged (four data sets per animal) and used for further analysis.

### Statistical analysis

Results are expressed as mean ± SEM. The differences among individual levels of EBF were analyzed using a repeated-measures one-way ANOVA with Tukey’s multiple comparison test, or using the Friedman test with Dunn’s multiple comparison test (for data sets without normal distribution); P < 0.05 was considered to be statistically significant. All statistical analyses were performed using GraphPad Prism 5.0 software (GraphPad, USA).

## Results

Myocardial hypoxia alone led to extensive myocardial injury sufficient to cause cardiogenic shock in four of five experimental animals. In one animal, an additional coronary artery occlusion was necessary to induce cardiogenic shock. Two animals were defibrillated (one and three attempts needed, respectively) because of ventricular fibrillation. All animals survived myocardial injury and successfully underwent all study procedures. The baseline values after the development of cardiogenic shock were: _P_CO 2.81 ± 0.34 L/min; SBP 60 ± 7 mmHg; LVEF 43 ± 3 %; and HR 94 ± 4 beats/min.

### Directly measured parameters

**Fig. 3:**
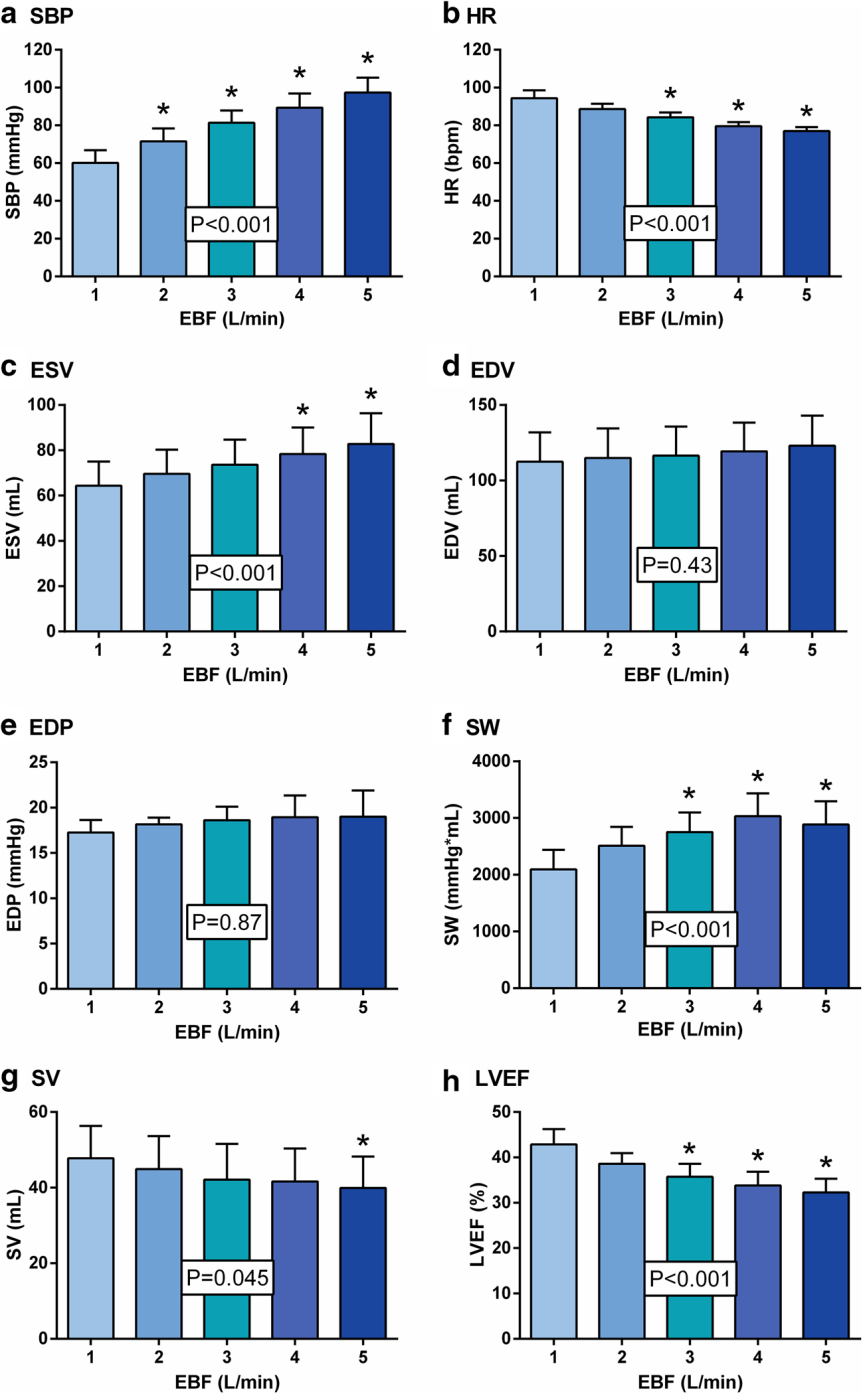
The effect of venoarterial extracorporeal membrane oxygenation blood flow on selected hemodynamic and left ventricular performance parameters in a porcine model of cardiogenic shock. *EBF* extracorporeal blood flow, *EDP* end-diastolic pressure, *EDV* end-diastolic volume, *ESV* end-systolic volume, *HR* heart rate, *LVEF* left ventricular ejection fraction, *SBP* systolic blood pressure, *SV* stroke volume, *SW* stroke work. *P < 0.05 compared with value at EBF 1 L/min

With increasing EBF, an increase in SBP was observed, from 60 ± 7 mmHg to 72 ± 7, 81 ± 6, 89 ± 7 and 97 ± 8 mmHg, respectively (EBF 1, 2, 3, 4 and 5 L/min; P < 0.001) (Fig. 3a); HR decreased from 94 ± 4 beats/min to 89 ± 3, 84 ± 3, 80 ± 2 and 77 ± 2 beats/min, respectively (EBF 1–5 L/min; P < 0.001) (Fig. 3b); _P_CO decreased from 2.81 ± 0.34 L/min to 2.49 ± 0.25, 2.21 ± 0.21, 1.84 ± 0.31 and 1.86 ± 0.53 L/min, respectively (EBF 1–5 L/min; P = 0.005) (Table 1); ESV increased from 64 ± 11 mL to 70 ± 11, 74 ± 11, 78 ± 12 and 83 ± 14 mL, respectively (EBF 1–5 L/min; P < 0.001) (Fig. 3c); EDV did not change significantly (112 ± 19, 115 ± 19, 116 ± 19, 119 ± 19 and 123 ± 20 mL, respectively; EBF 1–5 L/min, P = 0.43) (Fig. 3d); and although there was only a numerical increase in EDP from 17.2 ± 1.4 mmHg to 18.2 ± 0.7, 18.6 ± 1.5, 18.9 ± 2.4 and 19.0 ± 2.9 mmHg, respectively, these differences were not statistically significant (EBF 1–5 L/min; P = 0.87) (Fig. 3e). Finally, SW increased from 2096 ± 342 mmHg mL to 2510 ± 335, 2752 ± 346, 3031 ± 404 and 2884 ± 412 mmHg mL, respectively (EBF 1–5 L/min; P < 0.001) (Fig. 3f).

**Table 1:** The effect of venoarterial extracorporeal membrane oxygenation blood flow on selected hemodynamic parameters in a porcine model of cardiogenic shock

EBF	1	2	3	4	5	P
_LV_CO	4.31 ± 0.40	3.90 ± 0.47	3.49 ± 0.51*	3.21 ± 0.40*	2.99 ± 0.38*	<0.001
_P_CO	2.81 ± 0.34	2.49 ± 0.25	2.21 ± 0.21	1.84 ± 0.31*	1.86 ± 0.53*	<0.001
RecV	1.51 ± 0.24	1.40 ± 0.41	1.28 ± 0.42	1.38 ± 0.28	1.13 ± 0.37	0.83
RecF (%)	35.0 ± 4.8	36.0 ± 7.6	36.6 ± 7.9	42.8 ± 4.9	37.9 ± 10.7	0.57

Values of all parameters, with the exception of RecF, are expressed in L/min

*EBF* extracorporeal blood flow; _*LV*_ *CO* cardiac output, calculated from stroke volume (SV) and heart rate (HR) using the formula: _LV_CO = SV.HR; _*P*_ *CO* cardiac output, measured using a pulmonary artery catheter; *RecV* recirculation volume, calculated using the formula: RecV = _LV_CO − _P_CO; *RecF* recirculation fraction, calculated using the formula: RecF = (RecV/_LV_CO) × 100

* P < 0.05 compared with value at EBF 1 L/min

### Calculated parameters

With increasing EBF, a decrease in SV was observed, from 48 ± 9 mL to 45 ± 9, 42 ± 9, 41 ± 9 and 40 ± 8 mL, respectively (EBF 1–5 L/min, P = 0.045) (Fig. 3g); LVEF decreased from 43 ± 3 % to 39 ± 2 %, 36 ± 3 %, 34 ± 3 % and 32 ± 3 %, respectively (EBF 1–5 L/min; P < 0.001) (Fig. 3h); and _LV_CO decreased from 4.31 ± 0.40 L/min to 3.90 ± 0.47, 3.49 ± 0.51, 3.21 ± 0.40 and 2.99 ± 0.38 L/min, respectively (EBF 1–5 L/min; P < 0.001) (Table 1). The RecV and RecF remained comparable across the different EBF levels (Table 1).

## Discussion

The present study reports for the first time that in cardiogenic shock, EBF during VA ECMO affects invasively measured left ventricular performance parameters in a flow-dependent manner.

As a result of increasing EBF in the failing heart, we observed an increase in SBP and decrease in HR; these changes tended toward the normal values. In particular, elevation of low SBP in cardiogenic shock, together with optimizing tissue perfusion parameters, are the clearest and most easily measurable targets for ECMO therapy. However, it has been repeatedly demonstrated that increased SBP also leads to higher afterload, which may have a deleterious effect on the failing left ventricle [7]. Although a causal relationship between increased afterload and depressed left ventricular function in cardiogenic shock and VA ECMO therapy is more than likely, other factors (i.e., humoral factors or vegetative nervous system) may also contribute to this deleterious effect. Nevertheless, with increasing EBF and SBP, we observed an increase in ESV and decrease in SV and LVEF that resulted in decreased cardiac output.

Although EDV and EDP values increased numerically with increasing EBF, these differences did not reach statistical significance. However, EDV and EDP are markedly influenced not only by afterload but also by preload. At the highest EBF level, the right ventricle is almost entirely unloaded and the _P_CO rapidly decreases, which most likely lowers preload on the left ventricle. On the other hand, the preload on the failing left ventricle during VA ECMO may also be influenced by an increased fraction of bronchial circulation, and/or mitral and aortic regurgitation. In particular, aortic regurgitation is typically present in our experimental model due to the insertion of the PV-loop catheter through the aortic valve. However, based on echocardiography evaluation, this regurgitation appears to be of little significance.

To evaluate the effect of bronchial circulation, mitral regurgitation and aortic regurgitation on left ventricular preload, we also calculated values of the parameters of recirculation, RecV and RecF. Interestingly, these parameters were not significantly influenced by EBF levels. This observation may also indicate that recirculation plays a minor role in the development of distension of the overloaded left ventricle, with subsequent severe pulmonary edema, which is frequently observed in patients with severely depressed left ventricular function on high-flow VA ECMO.

Furthermore, we also observed elevation of SW with increased EBF (numerically highest at an EBF level of 4 L/min). This observation indicates that more energy needs to be expended by the left ventricle for each blood ejection at higher levels of EBF. It is unclear whether this increased energy demand is balanced by better coronary perfusion secondary to increased mean arterial pressure.

The occurrence of severe myocardial dysfunction as a result of VA ECMO has been acknowledged for many years. It has repeatedly been described in neonates with respiratory failure or with pulmonary hypertension [11–13], and similar findings were also observed in a lamb model [14]. Karr et al. [22] showed that impaired cardiac performance in infants is directly associated with ECMO therapy and that it is not a consequence of the primary lung disease. These studies are fully concordant with our results showing deterioration of left ventricular performance caused by VA ECMO in a flow-dependent manner. Shen et al. [16] have also shown that perfusion of coronary arteries by desaturated blood from the pulmonary circulation may play a role in the development of left ventricular dysfunction during VA ECMO. This is a highly clinically relevant issue, with risk for not only myocardial but also brain hypoxia. Monitoring coronary blood oxygen saturation is technically difficult in clinical settings; however, near-infrared spectroscopy oximetry offers continuous noninvasive measurement of brain oxygen saturation [23].

It could be argued that the myocardial left ventricular dysfunction that we attribute to ECMO-generated afterload was a consequence of global myocardial hypoxia due to desaturated blood from the pulmonary circulation. While this hypothesis cannot be fully ruled out, we consider it to be unlikely for several reasons: first, healthy animals with normal pulmonary function were used, and it is unlikely that severe pulmonary edema would develop within a few hours under our protocol; in addition, no signs of pulmonary edema (fluoroscopy, pulmonary mechanics) were observed throughout the study; second, we did not detect contractile dysfunction in nonaffected segments of the myocardium; and, finally, right ear SpO_2_ was maintained within normal limits.

Numerous studies have also focused on adult patients with severe myocardial dysfunction undergoing VA ECMO, usually with emphasis on the distension of the overloaded left ventricle, which is frequently associated with severe pulmonary edema and represents a serious clinical issue [7–10]. We did not observe the development of this critical condition in our study, even at the highest level of EBF. We also did not detect significant increases in EDV and EDP, although we observed numerically increased values in these parameters. There are several possible explanations for this discordance: first, we used an animal model of acute cardiogenic shock with the follow-up limited to approximately 150 min, and this interval may be too short for the development of left ventricular distension; second, left ventricular systolic function was not sufficiently impaired in our study to develop left ventricular distension; and, finally, the compliance of otherwise healthy left ventricle with acute regional hypoxic/ischemic injury is too low to allow distension.

Our results are consistent with the study by Aissaoui et al. [17] showing improvement in several echocardiographical parameters determining left ventricular performance in cardiogenic shock patients with decreasing VA ECMO flow.

The present study had several limitations. First, we used only five animals; however, the present research was a pilot study using an alternative model of cardiogenic shock that increases the survival rate immediately following extensive acute myocardial injury. Second, the extent of myocardial injury was different due to individual coronary anatomy; on the other hand, all animals fulfilled the defined criteria of cardiogenic shock with signs of tissue hypoperfusion. Third, we used young and otherwise healthy animals; therefore, our experimental settings simulate clinical conditions in patients with cardiogenic shock caused by acute myocardial infarction rather than severe myocarditis or advanced dilated cardiomyopathy.

## Conclusions

Our results demonstrate that increasing EBF in cardiogenic shock during VA ECMO may cause impairment of left ventricular performance in a flow-dependent manner. These data indicate that the optimal VA ECMO flow in cardiogenic shock should be as low as possible to allow adequate tissue perfusion.
